# Angina Pectoris

**Published:** 1912-04-13

**Authors:** Henry J. Nash

**Affiliations:** London University; late Coach Anatomy and Physiology, Cambridge University. 291 Gwendwr Road, West Kensington, London


ANGINA PECTORIS.

*To the Editor of* The Hospital.

Sir,—Will you kindly permit me space to touch upon a condition of " angina pectoris " which is seldom met with, and one which would properly be included under the heading of "Vague Anginal Forms"? I refer to angina without pain.

In recent discussions published in the *British Medical Journal* dealing with the subject of cardiac asthma, only one contributor appeared to be fully aware that a vascular spasm of angina pectoris, instead of being one of pain, might give way to difficulty of breathing, which latter, in the case of angina, would be cardiac asthma. Dr. W. Bezly Thorne, in his manual on " The Schott Methods of the Treatment of Chronic Diseases of the Heart," recognises one case. He refers to it as " Angina sine Dolore."

In these conditions of angina without any manifestation of pain, the only symptoms which are apparent are a feeling of præcordial distress and impending death.—Yours, etc., Henry J. Nash,

291 Gwendwr Road, West Kensington, London, April 2.

London University; late Coach Anatomy and Physiology, Cambridge University.

